# Disialoganglioside GD2 Expression in Solid Tumors and Role as a Target for Cancer Therapy

**DOI:** 10.3389/fonc.2020.01000

**Published:** 2020-07-07

**Authors:** Bassel Nazha, Cengiz Inal, Taofeek K. Owonikoko

**Affiliations:** ^1^Department of Hematology and Medical Oncology, Emory University School of Medicine, Atlanta, GA, United States; ^2^Salem Veterans Affairs Medical Center, Salem, VA, United States

**Keywords:** GD2, ganglioside, monoclonal antibody, cancer therapy, neuroblastoma, small cell lung cancer, dinutuximab, clinical trials

## Abstract

Gangliosides are carbohydrate-containing sphingolipids that are widely expressed in normal tissues, making most subtypes unsuitable as targets for cancer therapy. However, the disialoganglioside GD2 subtype has limited expression in normal tissues but is overexpressed across a wide range of tumors. Disialoganglioside GD2 can be considered a tumor-associated antigen and well-suited as a target for cancer therapy. Disialoganglioside GD2 is implicated in tumor development and malignant phenotypes through enhanced cell proliferation, motility, migration, adhesion, and invasion, depending on the tumor type. This provides a rationale for targeting disialoganglioside GD2 in cancer therapy with the development of anti-GD2 monoclonal antibodies and other therapeutic approaches. Anti-GD2 monoclonal antibodies target GD2-expressing tumor cells, leading to phagocytosis and destruction by means of antibody-dependent cell-mediated cytotoxicity, lysis by complement-dependent cytotoxicity, and apoptosis and necrosis through direct induction of cell death. Anti-GD2 monoclonal antibodies may also prevent homing and adhesion of circulating malignant cells to the extracellular matrix. Disialoganglioside GD2 is highly expressed by almost all neuroblastomas, by most melanomas and retinoblastomas, and by many Ewing sarcomas and, to a more variable degree, by small cell lung cancer, gliomas, osteosarcomas, and soft tissue sarcomas. Successful treatment of disialoganglioside GD2-expressing tumors with anti-GD2 monoclonal antibodies is hindered by pharmacologic factors such as insufficient antibody affinity to mediate antibody-dependent cell-mediated cytotoxicity, inadequate penetration of antibody into the tumor microenvironment, and toxicity related to disialoganglioside GD2 expression by normal tissues such as peripheral sensory nerve fibers. Nonetheless, anti-GD2 monoclonal antibody dinutuximab (ch14.18) has been approved by the U.S. Food and Drug Administration and dinutuximab beta (ch14.18/CHO) has been approved by the European Medicines Agency for the treatment of high-risk neuroblastoma in pediatric patients. Clinical trials of anti-GD2 therapy are currently ongoing in patients with other types of disialoganglioside GD2-expressing tumors as well as neuroblastoma. In addition to anti-GD2 monoclonal antibodies, anti-GD2 therapeutic approaches include chimeric antigen receptor T-cell therapy, disialoganglioside GD2 vaccines, immunocytokines, immunotoxins, antibody–drug conjugates, radiolabeled antibodies, targeted nanoparticles, and T-cell engaging bispecific antibodies. Clinical trials should clarify further the potential of anti-GD2 therapy for disialoganglioside GD2-expressing malignant tumors.

## Introduction

**Figure 1:**
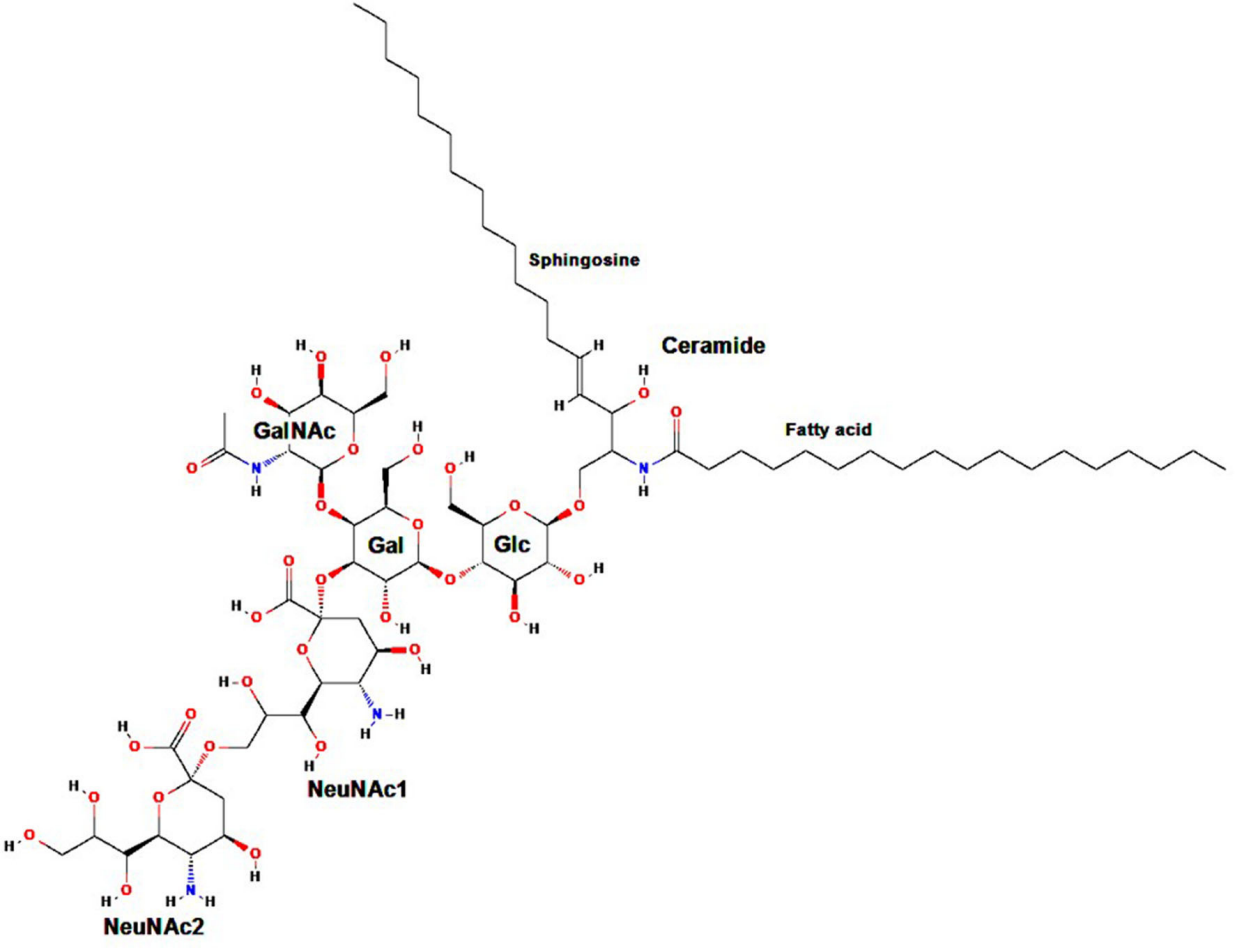
Molecular structure of GD2. Gal, galactose; GalNAc, N-acetylgalactosamine; Glc, glucose; NeuNAc, N-acetylneuraminic acid. Adapted from: Ganglioside GD2. NIH. U.S. National Library of Medicine. National Center for Biotechnology Information. Available at: https://pubchem.ncbi.nlm.nih.gov/compound/53481124.

Gangliosides are carbohydrate-containing sphingolipids (glycosphingolipids) composed of a ceramide (usually sphingosine, a long-chain amino alcohol, attached by an amide group to a fatty acid core that varies in chain length from C18 to C20) bound to N-acetylneuraminic acid (Neu5Ac) or another sialic acid (an acidic carbohydrate with a nine-carbon backbone) linked to one or more monosaccharide units (1–3). Standard ganglioside nomenclature begins with the letter G (“ganglioside”), followed by the letter M, D, T, Q, P, H, or S referring to mono-, di-, tri-, quatra- (tetra-), penta-, hexa-, and septasialogangliosides, respectively, based on the number of sialic acid residues (2–4), and ending with the numbers 1, 2, or 3 indicating the order of ganglioside migration on thin layer chromatography (5). As an example, the ganglioside GD2 subtype comprises two sialic acid residues (i.e., a disialoganglioside) linked to three monosaccharide units (Figure 1).

Intracellular synthesis of gangliosides begins with formation of the ceramide core, followed by addition of the monosaccharide unit(s) and translocation to the plasma membrane (6, 7). Attached to the outer plasma membrane by their ceramide moiety, gangliosides interact laterally with membrane proteins and other membrane lipids to regulate the responsiveness of signaling molecules and to function as mediators and modulators of signal transduction (8). Their monosaccharide units extend into the extracellular space, where they have antigenic properties and facilitate cell–cell recognition and adhesion (8–10).

Gangliosides such as GM3, GM2, GM1, and GD1 are expressed across many normal human tissues (6, 10–12) and appear to have multiple biological functions, particularly cell recognition and regulation of membrane-bound signaling proteins such as epidermal growth factor receptor and vascular endothelial growth factor receptor (10, 13). In contrast, expression of disialoganglioside GD2 in normal tissues is limited essentially to the central nervous system, peripheral sensory nerve fibers, dermal melanocytes, lymphocytes, and mesenchymal stem cells (12, 14, 15). Although disialoganglioside GD2 appears to participate in cell signaling, its function in normal cellular physiology has not been fully elucidated and therefore is not well-understood (16). In cancer, however, disialoganglioside GD2 contributes to enhanced tumor cell proliferation, motility, migration, adhesion, and invasion, depending on the tumor type, and confers resistance to apoptosis (12, 17–20).

This review will focus on disialoganglioside GD2 as a target for cancer therapy and various approaches under investigation aimed at GD2-expressing tumors.

## Disialoganglioside GD2 Antigen as a Therapeutic Target in Cancer

### Restricted Tumor Expression

Although many gangliosides are expressed by a wide range of human tumor cells, generally they are not considered potential targets for cancer therapy because of their extensive expression by normal tissues (6, 10, 11, 21). Given its very limited expression in normal human tissues and extensive expression by a number of tumors, disialoganglioside GD2 can be considered a tumor-associated antigen (14, 22, 23) and thus is valuable as a primary target for cancer immunotherapy (15). Indeed, the U.S. National Cancer Institute ranked disialoganglioside GD2 twelfth among 75 potential targets for anti-cancer therapy in 2009 based on potential therapeutic effect, degree of expression, immunogenicity, and percentage of antigen-specific cells (24).

#### Neuroblastoma

GD2 is the major ganglioside present in human neuroblastoma cell lines, is synthesized in large quantity by the great majority of primary untreated neuroblastomas and is detectable in plasma and tumor tissue of patients with neuroblastoma regardless of the disease stage (25, 26). More rapid progression of neuroblastoma and lower patient survival appear to be associated with higher circulating tumor-derived disialoganglioside GD2 levels at the time of diagnosis (27); circulating GD2 levels decrease in response to therapy and increase again in patients with recurrent disease (28).

GD2 expression by neuroblastoma cells can be detected using different technologies including enzyme-linked immunosorbent assay (ELISA), immunoperoxidase staining of frozen tissues, and high-pressure liquid chromatography/tandem mass spectrometry (25, 29). Schulz et al. identified GD2 serum levels >26 ng/mL (mean of controls ±3 S.D.) by ELISA in 21 of 23 pre-chemotherapy neuroblastoma patients (91.3%), six of whom had levels >1,000 ng/mL (30). Two of the six patients presented with stage III disease and four with stage IV disease. Of six patients with stage I or II disease, none had GD2 serum levels >100 ng/mL. Disialoganglioside GD2 was expressed at high tissue concentrations in 100% of 36 cases of primary untreated neuroblastoma as reported by Wu et al. (31). Untreated neuroblastomas were of all stages (I–IV) and thus GD2 expression was stage-independent. Similarly, Cheung et al. reported a series of eight cases of neuroblastoma, all of which expressed GD2 as determined by *in vitro* immunostaining and/or *in vivo* radioimaging (32). Schengrund and Shochat identified disialoganglioside GD2 in 45 of 53 childhood neuroblastomas (84.9%) (33). In the series reported by Sariola et al., 28 of 30 pediatric neuroblastomas (93.3%) were GD2-positive (26). Yeh et al. compared radioimmunoscintigraphy with an ^131^I-radiolabeled anti-GD2 mAb (^131^I-3F8), ^131^I-MIBG (metaiodobenzylguanidine), and other imaging modalities in 42 consecutive patients with stage III or IV neuroblastoma (34). ^131^I-3F8 identified primary and metastatic neuroblastoma with excellent sensitivity and specificity. Surgical resection and subsequent histopathologic examination in nine patients revealed seven who were ^131^I-3F8 scan-positive and all tumors were confirmed as neuroblastoma; the two tumors that were ^131^I-3F8 negative were diagnosed as ganglioneuromas, one of which had microscopic foci of neuroblastoma. Zang et al., using immunohistology techniques, found >50% GD2-positive cells in 5 of 5 frozen tissue specimens of human neuroblastoma (21). More recently, cytomorphologic examination with light microscopy plus multi-parametric flow cytometry with a panel that included disialoganglioside GD2 had greater sensitivity and specificity than cytomorphology alone for the detection of metastatic neuroblastoma in bone marrow (35).

#### Small Cell Lung Cancer

##### Cell surface expression

Gangliosides GM2 and GM1 are expressed by almost all subsets of lung cancer cell lines, whereas disialoganglioside GD2 is found characteristically in SCLC lines but is not expressed at all or is expressed at only very low levels by non-small cell lung cancer (NSCLC) lines (14). Disialoganglioside GD2 has been detected in cultured SCLC cell lines as well as in peripheral blood and bone marrow samples of patients with SCLC (14, 36, 37). Disialoganglioside GD2 expression is also much higher in SCLC cell lines than in normal lung cell lines (25, 36). However, quantitative data regarding expression of disialoganglioside GD2 by SCLC cells currently are limited.

Cheresh et al. detected disialoganglioside GD2 on both cultured cell lines and frozen biopsy specimens of human SCLC, using an ELISA assay and two anti-GD2 mAbs as molecular probes (36). Conversely, Zhang et al., using immunohistochemical analyses, identified >50% GD2-positive cells in 0 of 6 frozen tissue specimens of human SCLC (21). Grant et al. evaluated the ability of an ^131^I-radiolabeled anti-GD2 mAb to target tumor sites in 10 patients with untreated or recurrent/progressive SCLC (38). These radionuclide scans along with single photon emission tomography fusion image identified all known tumor sites except for a small brain metastasis in one patient. Yoshida et al. analyzed the expression of disialoganglioside GD2 across 44 lung cancer cell lines using flow cytometry and determined that GD2 was found characteristically in SCLC cell lines but was absent in or only minimally expressed by NSCLC lines, suggesting that GD2 may be a good therapeutic target in SCLC (14). Because disialoganglioside GD2 synthesis is dependent on GD2/GM2 synthase, Chen et al. conducted a pilot study of patients with SCLC and detected GD2/GM2 synthase in the peripheral blood of those with high expression in six SCLC cell lines (37). However, these results could not be confirmed in a prospective study by the authors, and they concluded that GD2/GM2 synthase is not a reliable biomarker for SCLC (37).

##### Association between GD2 expression and malignant phenotypes

Disialoganglioside GD2 promotes malignant phenotypes (20) such as increased proliferation, growth, migration, and invasion activity of SCLC tumor cells (17, 20), but the exact mechanisms underlying these effects are not well-known (17, 20). It has been speculated that GD2 may promote malignant phenotypes in SCLC by recruiting ASC amino-acid transporter (ASCT2)—a major glutamine transporter that has an essential role in tumor growth and progression—to lipid rafts (glycolipid-enriched domains on the outer leaflet of the plasma membrane bilayer), leading to enhanced glutamine uptake through ASCT2 (12, 20). In addition, overexpression and shedding of disialoganglioside GD2 by tumor cells into the tumor microenvironment can modulate normal cells present, supporting angiogenesis and tumor immune evasion (immunologic escape) while inhibiting cellular immune responses of lymphocytes (T-cell apoptosis, for example) and antigen-presenting cells (7, 39, 40). In support of a critical role for disialoganglioside GD2 in cell proliferation, anti-GD2 mAbs (220-51 [mouse IgG3], KM666 [mouse IgG3], and KM1138 [mouse-human chimeric mAb]) suppressed the proliferation of GD2-expressing SCLC cells (14, 17) and induced apoptosis of SCLC cells via activation of caspases, a group of proteases with crucial roles in programmed cell death (17).

#### Other Tumors

Studies have shown that GD2 is expressed across a range of tumors. For example, Zhang et al. identified GD2-positive cells in 6 of 10 frozen tissue specimens (60%) of human melanoma and 5 of 9 specimens (55.6%) of sarcoma (21). Dobrenkov et al. found that the expression of disialoganglioside GD2 was highly prevalent (≥70%) in pediatric melanoma and osteosarcoma, with uniformly strong intensity on immunohistochemistry staining in osteosarcoma, but did not exceed 50% in other pediatric solid tumors including rhabdomyosarcoma, Ewing sarcoma, and desmoplastic small round cell tumors (41). The authors concluded that disialoganglioside GD2 is a promising target for antibody-based therapy in pediatric solid tumors.

##### Melanoma

Disialoganglioside GD2 is the main ganglioside expressed on M21 melanoma cells (42). Cheresh et al. demonstrated with indirect immunofluorescence and scanning immunoelectron microscopy that disialoganglioside GD2 preferentially localizes into substrate-associated microprocesses originating from the plasma membrane of M21 melanoma cells and facilitates their attachment to fibronectin and other adhesive glycoproteins in the extracellular matrix (42, 43). Ohmi et al. compared the phenotypes of ganglioside-expressing melanoma cells and determined that disialoganglioside GD2 played a major role in increased cell growth, cell adhesion to collagen, and cell spreading, but did not play as large a role in cell migration velocity and invasion activity compared with gangliosides GD3, GM2, GM1, and GM3 (44). These results suggest that disialoganglioside GD2 may contribute to the fixation of melanoma cells at sites of metastasis. Cheresh and Pierschbacher found the relative surface expression of disialoganglioside GD2 antigens by 47 human melanoma cell lines varied from 80.5 to 100% depending on the anti-GD2 mAb and the analytic method used (43). Tsuchida et al. studied the ganglioside composition of melanoma tissue obtained by biopsy and identified disialoganglioside GD2 in 2.4% of 26 specimens obtained from skin and subcutaneous tissue, compared with 13.8% of 12 cultured melanomas derived from skin and subcutaneous tissue (45).

##### Ewing sarcoma

Ewing sarcoma has been studied for disialoganglioside GD2 expression, and the results ranged from no detectable surface expression to diffuse and/or intense staining in some tumors (46). More specifically, GD2 expression levels ranged from 40 to 90% from diagnostic biopsy samples of Ewing sarcoma (47). Kailayangiri et al. detected disialoganglioside GD2 expression by immunofluorescence staining in 10 of 10 Ewing sarcoma cell lines and 3 of 3 primary cell cultures, concluding that surface expression of disialoganglioside GD2 is a characteristic of Ewing sarcoma and that GD2 provides an appropriate target antigen for therapeutic strategies to eradicate micrometastases and reduce the risk of recurrence in patients with high-risk disease (48). Accordingly, disialoganglioside GD2 is a potential therapeutic target in Ewing sarcoma.

##### Osteosarcoma

Roth et al. used immunohistochemistry analysis to study the expression of disialoganglioside GD2 in 44 patient-derived osteosarcoma cell lines and observed higher staining intensity in specimens obtained at tumor recurrence compared with specimens obtained at initial resection (49). This finding suggests that disialoganglioside GD2 plays a role in chemotherapy resistance and/or tumor progression of osteosarcoma. Zhu et al. identified a synergistic effect of an anti-GD2 mAb and cisplatin compared with their individual effects on osteosarcoma cells; the results suggested that cisplatin plus an anti-GD2 mAb could be an effective therapeutic strategy for osteosarcoma (50). Heiner et al. tested fresh-frozen samples of osteosarcoma from 17 patients for the presence of GD2 by immunofluorescence following exposure to anti-GD2 mAb and found that 15 of 17 tumors (88.2%) showed strong reactivity and high intensity of 3+ or 4+ in more than 95% cells stained; the two tumors with weak intensity of staining proved to be variants of malignant fibrous histiocytoma (51). Butch et al. used positron emission tomography (PET) to evaluate the ability of a novel radiolabeled, humanized anti-GD2 mAb ([^64^Cu]Cu-Bn-NOTA-hu14.18K322A) to detect GD2 expression in a mouse model of osteosarcoma *in vivo* (52). Tumor uptake of the radiolabeled mAb was 7-fold higher in modestly GD2-expressing osteosarcomas than in a GD2-negative tumor, and PET scan could identify lesions as small as 29 mm^3^ in a 34% GD2-positive model of metastatic osteosarcoma of the lung. These results support the utility of disialoganglioside GD2 imaging with PET to measure GD2 expression in osteosarcoma and thus maximize the clinical impact of anti-GD2 targeted therapy.

##### Soft tissue sarcoma

Chang et al. studied tissue from 56 freshly frozen human sarcomas by immunohistochemical staining and found that 52 (93%) expressed disialoganglioside GD2 (53). The intensity of GD2 expression varied among sarcoma types and was weakest for more aggressive tumors (embryonal rhabdomyosarcoma and synovial sarcoma). Supporting this observation, Saraf et al. identified disialoganglioside GD2 expression in only 25% of 16 pediatric rhabdomyosarcoma samples (54).

##### Glioma

Disialoganglioside GD2 is expressed by some gliomas (25, 55) including glioblastomas (56), and GD2 expression by these tumors is associated with increased cell growth, mobility, and invasiveness (57). Longee et al. used an anti-GD2 mAb and identified disialoganglioside GD2 expression in 80% of malignant glioma cell lines (*N* = 20) and tumor biopsies of malignant glioma (*N* = 30) (55).

##### Retinoblastoma

Disialoganglioside GD2 is expressed on the cell surface of retinoblastomas and may be detectable in the serum (58), bone marrow (59), and cerebrospinal fluid (60) of patients with extraocular dissemination. Chantada et al. identified bone marrow invasion by retinoblastoma in 11 of 27 patients with extraocular disease (59). Disialoganglioside GD2 was expressed intensely in the bone marrow of all nine patients whose marrow specimens were examined immunocytologically. Sujjitjoon et al. immunohistochemically examined surgical specimens from eight patients with retinoblastoma and found all to be strongly positive for disialoganglioside GD2 expression (61). Andersch et al. studied the expression of disialoganglioside GD2 in 11 retinoblastoma cell lines and found GD2 to be expressed by all (62).

##### Breast and bladder cancer

Disialoganglioside GD2 is also expressed by a small number of tumors not of neuroectodermal origin. For example, GD2 has been identified as a marker for breast cancer stem cells in a small proportion of human breast cancer cell lines (9). Orsi et al. found that breast cancer cells obtained by surgical resection of tumor from 63 previously untreated patients were positive for disialoganglioside GD2 in 37 cases (59%); the percentage of cells that stained positive for GD2 by immunohistochemistry in these specimens ranged between 3 and 100% (mean: 52%) (63). In addition, the majority of aggressive breast cancer subtypes (triple-negative and metaplastic specimens) tested positive for disialoganglioside GD2. Mansoori et al. showed higher GD2 expression in breast cancer samples from patients with advanced histological grade (*P* = 0.02), lymph node invasion (*P* = 0.04), larger tumor size (*P* = 0.04), and older age (*P* = 0.04) (64). Finally, Vantaku et al. reported that high-grade bladder cancer tissue and cell lines express disialoganglioside GD2 at a level higher than that of low-grade bladder cancer, and GD2-expressing muscle-invasive bladder cancer cells proliferated more rapidly than GD2-negative cells (65). The authors considered disialoganglioside GD2 expression to be a biomarker for aggressive bladder cancer.

## Targeting Disialoganglioside GD2-Expressing Tumors

In addition to neuroblastoma, anti-GD2 targeted therapy currently is being investigated as treatment of a number of GD2-expressing tumors including SCLC in adults, osteosarcoma in children and adults, glioma, soft tissue sarcoma, Ewing sarcoma, retinoblastoma, and melanoma.

### Anti-GD2 mAbs

#### Proposed Mechanisms of Action in Cancer

**Figure 2:**
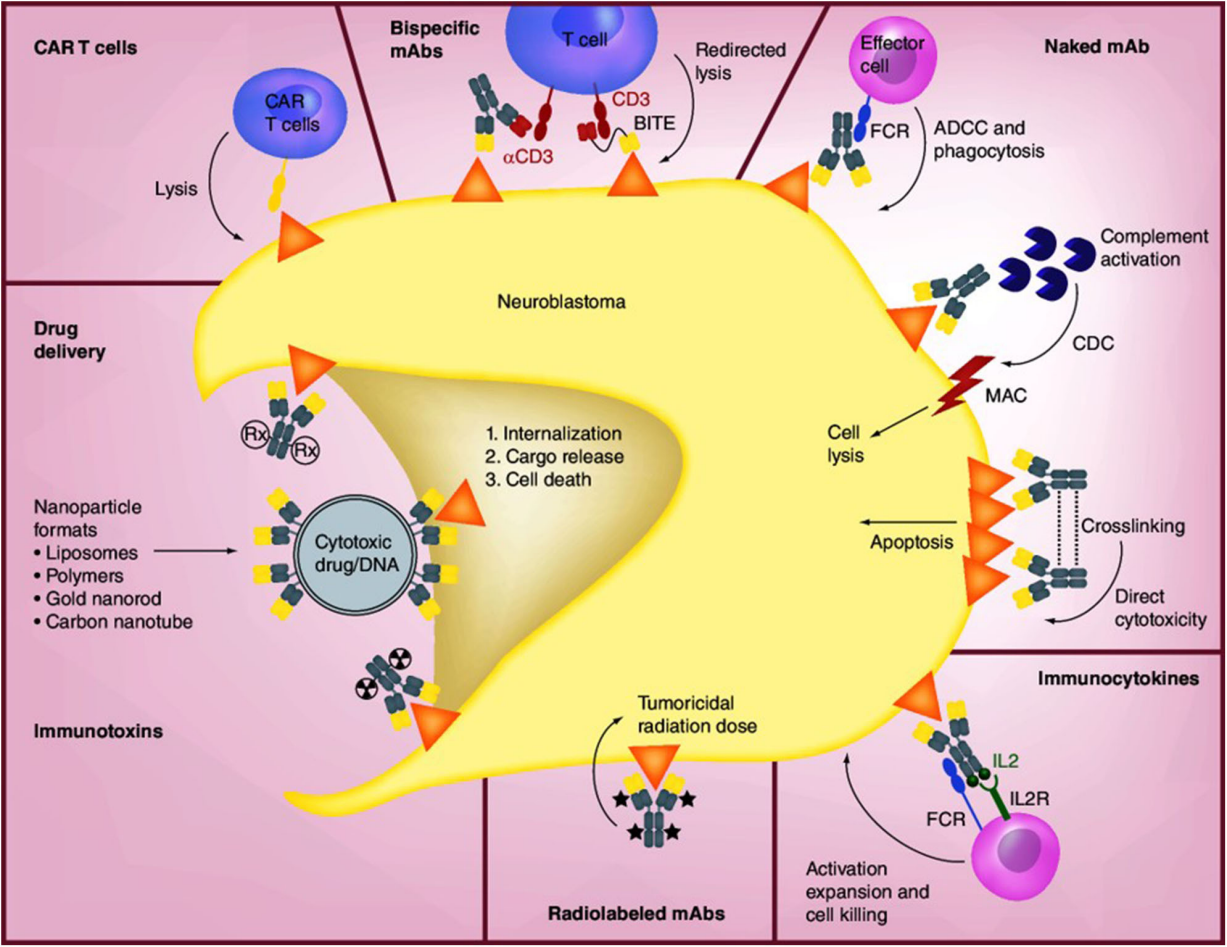
Mechanisms of anti-GD2 mAbs and other therapeutic approaches [Source: Perez Horta et al. (66)]. The binding of anti-GD2 mAbs to GD2 on the surface of neuroblastoma cells results in three proposed antitumor effects: (1) attraction of NK cells, granulocytes, and other FCR-expressing effector cells to promote ADCC, together with the recruitment of macrophages and monocytes to mediate phagocytosis; (2) induction of CDC by binding of the mAb to complement component 1q (C1q), followed by activation of the complement cascade and transport of MAC to the plasma membrane of the tumor cell; and (3) direct cytotoxicity through the initiation of apoptosis. The concentration of IL-2-based immunocytokines at the tumor promotes activation of tumoricidal effector cells via their IL-2R and FC receptors. Radiolabeled mAbs serve the dual function of tumor radioimmunodetection and delivery of tumoricidal doses of radiation to tumor cells. Immunotoxins and drug conjugates can deliver various toxic agents to tumor cells, followed by internalization and release of the toxic agent and cell death. CAR-T cells are engineered *ex vivo* to recognize the tumor antigen and promote tumor cell lysis. Bispecific mAbs are available in a variety of forms such as hybrid bifunctional mAbs with two different antigen-specific regions or as BITE with the primary objective of redirecting T cells for tumor lysis by engaging tumor antigen and costimulatory molecules such as CD3. ADCC, antibody-dependent cell-mediated cytotoxicity; BITE, bispecific T-cell engagers; CAR, chimeric antigen receptor; CDC, complement-dependent cytotoxicity; FCR, FC-receptor; IL2, interleukin-2; IL2R, interleukin-2 receptor; mAb, monoclonal antibody; MAC, membrane attack complex. Reproduced with permission as agreed by Future Medicine Ltd.

Anti-GD2 mAbs have three proposed mechanisms of action (MOA) against disialoganglioside GD2-expressing tumor cells: (1) induction of phagocytosis by macrophages and destruction of tumor cells by natural killer (NK) cells and granulocytes via antibody-dependent cell-mediated cytotoxicity (ADCC), (2) lysis of tumor cells via complement-dependent cytotoxicity (CDC), and (3) direct induction of cell death due to specific binding of anti-GD2 mAbs to GD2 (Figure 2) (66–68). In ADCC—the primary MOA—tumor-bound anti-GD2 mAbs engage Fcγ receptors on the surface of NK cells and granulocytes, followed by the release from these cells of cytotoxic granules (serine proteases, or granzymes [specifically, granzyme B]) and cytokines (particularly perforin, a glycoprotein that creates pores in targeted cell membranes) (69), causing Fc-dependent phagocytosis and lysis of tumor cells (68, 70). CDC is induced through binding of the serine protease complex C1 to the Fc domains of two or more mAbs binding to antigens expressed on tumor cells (70). This classic complement pathway results in an activation cascade causing the membrane attack complex (MAC) to disrupt the target cell. In mAb-mediated CDC, it is hypothesized that antibody-coated tumor cells activate the complement system, which is followed by cancer cell lysis via MAC accumulation or phagocytosis of opsonized cancer cells by macrophages and granulocytes.

Anti-GD2 mAbs also may induce cell death directly without involving immune mechanisms, combining features of apoptosis and necrosis in GD2-positive tumor cell lines but not in GD2-negative tumors (67, 71). Direct induction of mAb-mediated tumor cell death occurs in a dose-dependent manner, with the strongest cytotoxic effects observed in tumor cells with the highest expression of GD2. The process appears to involve alteration of mitochondrial membrane potential, change in plasma membrane permeability, and induction of apoptotic volume decrease (apoptotic whole-cell shrinkage, a major hallmark of apoptotic cell death). In addition, anti-GD2 mAbs inhibit attachment of circulating malignant cells to protein components of the extracellular matrix (43), potentially representing a fourth MOA.

#### Clinical Availability and Selection

Therapeutic mAbs against disialoganglioside GD2 can be generated using murine, chimeric, humanized, and human antibody constructs (72, 73). Development of anti-ganglioside agents generally and anti-GD2 agents specifically requires engineering GD2 mAbs. The major pharmacologic obstacles to successful treatment of GD2-expressing tumors with anti-GD2 mAbs include insufficient antibody affinity to mediate ADCC, inadequate penetration of antibody into the microenvironment of the tumor, and toxicity related to disialoganglioside GD2 expression by normal tissues (particularly peripheral nerves) (74). The murine IgG3 monoclonal antibody 3F8 was developed in 1985 and was the first specific anti-GD2 antibody (32), followed by the murine IgG3 mAb 14.18 and then the chimeric IgG1 murine-human mAb ch14.18 (75). Chimeric and humanized anti-GD2 mAbs are less immunogenic, exhibit longer half-lives, and more efficiently promote effector functions than murine mAbs (73, 76).

Preclinical pharmacologic studies characterized the binding and activity profiles of ch14.18 and 14.G2a, a murine anti-GD2 mAb. Mujoo et al. and Mueller et al. reported that both of these antibodies had similar binding affinity for disialoganglioside GD2 and demonstrated the ability to mediate ADCC and CDC (77, 78). Mujoo et al. demonstrated highly specific and avid binding of ch14.18 to disialoganglioside GD2 expressed by a variety of tumor cell lines of neuroectodermal origin, including eight different neuroblastoma cell lines and several melanoma, glioblastoma, and SCLC lines (25). Earlier work had suggested a functional role for disialoganglioside GD2 in helping to facilitate the attachment of neuroblastoma and melanoma cells to the extracellular matrix (42, 43). For example, Mueller et al. reported that the number of GD2 binding sites on cell lines correlated with levels of antibody binding (78). These findings made anti-GD2 antibodies appealing candidates for further evaluation.

##### Dinutuximab

Dinutuximab (ch14.18; Unituxin®, United Therapeutics Corporation) is produced in mouse myeloma cell line SP2/0 (75). Dinutuximab used together with granulocyte-macrophage colony-stimulating factor (GM-CSF), interleukin-2 (IL-2), and isotretinoin was licensed in March 2015 by the U.S. Food and Drug Administration (FDA) “for the treatment of pediatric patients with high-risk neuroblastoma who achieve at least a partial response to prior first-line, multi-agent, multimodality therapy” (79). Dinutuximab is the first FDA-approved treatment specifically for high-risk neuroblastoma. Dinutuximab was also approved in the EU in August 2015 for the same indication.

##### Dinutuximab beta

Dinutuximab beta (ch14.18.CHO; Qarziba®, EUSA Pharma), is a murine-human chimeric anti-GD2 mAb produced in Chinese hamster ovary (CHO) cells (75). CHO cells for mAb expression provide a glycosylation pattern that includes only small amounts of the sialic acid N-glycolylneuraminic acid, avoiding its rapid clearance by human autoantibodies, and precludes the potential murine virus contamination associated with SP2/0 cells (68). Dinutuximab beta was approved by the European Commission in May 2017 “for the treatment of high-risk neuroblastoma in patients aged 12 months and above who have previously received induction chemotherapy and achieved at least a partial response followed by myeloablative therapy and stem cell transplantation.” It is also approved in the EU for patients “with a history of relapsed or refractory neuroblastoma with or without residual disease.” Dinutuximab beta currently is investigational in the US and is being evaluated with IL-2 and isotretinoin for treatment of neuroblastoma (80). In the pivotal phase III trial, however, the investigators noted that there was “no evidence that addition of subcutaneous IL-2 to immunotherapy with dinutuximab beta...improved outcomes in patients with high-risk neuroblastoma” but IL-2 “was associated with greater toxicity than dinutuximab beta alone” (81).

##### Investigational anti-GD2 mAbs

A number of investigational anti-GD2 mAbs currently are being evaluated in clinical trials, primarily for neuroblastoma, and these mAbs are summarized in Table 1. For example, humanized 3F8 (3F8 humanized; hu3F8; naxitamab) mAb has higher affinity than dinutuximab for disialoganglioside GD2 antigen and superior ADCC (but not CDC) than murine 3F8 mAb (82). Humanized anti-GD2 antibodies have been developed to diminish the potential for patients to generate human anti-murine antibodies, which might block the antitumor activity of anti-GD2 antibodies (75). In a phase I clinical trial in patients with resistant or recurrent neuroblastoma, hu3F8 had low immunogenicity, produced modest toxic effects, and possessed considerable activity against neuroblastoma. In addition, multimerization and pegylation of single-chain fragment variables (scFv) of anti-GD2 antibodies were shown to significantly increase circulation time compared with that of monomeric scFv in the blood and to increase tumor penetration of the anti-GD2 antibodies in a syngeneic GD2-positive mouse cancer model (83). Furman et al. reported the results of a non-randomized phase II trial in which the humanized disialoganglioside GD2 mAb hu14.18K322A was coadministered with six courses of induction chemotherapy in 42 children with newly diagnosed high-risk neuroblastoma (84) Thirty two patients (76.2%) experienced at least a partial response following two courses of chemoimmunotherapy, together with a median reduction of volume of the primary tumor of 76%. The authors concluded that “Adding hu14.18K322A to induction chemotherapy produced early partial response or better in most patients, reduced tumor volumes, improved cancer-specific survival at the end of induction, and yielded an encouraging 2-year event-free survival.”

**Table 1:** ch14.18, ch14.18CHO, and Investigational anti-GD2 mAbs^*^.

**Antibody**	**Description**	**Features**
**Approved**		
ch14.18 (dinutuximab)	Chimeric (murine-human) IgG1 mAb produced in murine myeloma SP2/0 cell line	FDA- and EMA-approved for neuroblastoma^†^
ch14.18/CHO (dinutuximab beta)	Chimeric (murine-human) IgG1 mAb produced in CHO cells	EMA-approved for neuroblastoma^‡^
**Investigational**		
14.18	Murine IgG1 mAb	Lower ADCC than 14.G2a
14.G2a	Murine IgG2a mAb	Used to generate ch14.18
3F8	Murine IgG3 mAb	Large experience as single agent and in combinations
Hu3F8	Humanized 3F8 mAb	Less complement activation than 3F8
^131^I-3F8	Murine mAb fused with iodine 131	Radioimmunoconjugate with radioimaging and radioimmunotherapeutic properties
Hu14.18-IL-2	Humanized 14.18 mAb fused with IL-2	Clinical trials of fusion version with IL-2
Hu14.18K322A	Point mutation in hu14.18 (biologically modified from 14.G2a)	Designed to reduce complement activation and subsequent painful side effects
ME36.1	Murine mAb class switched to IgG1 and IgG2a	Cross reacts with GD3
8B6	mAb that binds to *O*-acetyl-GD2 antigen	May reduce painful side effects
L72	Fully human IgM mAb	Produced by EBV-transformed cell lines

*ADCC, antibody-dependent cellular cytotoxicity; CHO, Chinese hamster ovary; EBV, Epstein-Barr virus; EMA, European Medicines Agency; FDA, U.S. Food and Drug Administration; mAb, monoclonal antibody*.

* *Adapted from Keyel (75)*.

† *Indicated, in combination with granulocyte-macrophage colony-stimulating factor (GM-CSF), interleukin-2 (IL-2), and 13-cis-retinoic acid (RA), for the treatment of pediatric patients with high-risk neuroblastoma who achieve at least a partial response to prior first-line multiagent, multimodality therapy*.

‡ *Indicated for the treatment of high-risk neuroblastoma in patients aged 12 months and above, who have previously received induction chemotherapy and achieved at least a partial response, followed by myeloablative therapy and stem cell transplantation, as well as patients with history of relapsed or refractory neuroblastoma, with or without residual disease. Prior to the treatment of relapsed neuroblastoma, any actively progressing disease should be stabilized by other suitable measures. In patients with a history of relapsed/refractory disease and in patients who have not achieved a complete response after first line therapy, Qarziba should be combined with interleukin-2 (IL-2)*.

The severe nociceptive pain that commonly accompanies treatment with anti-GD2 mAbs may limit the dose that can be infused and, as a result, potentially reduce clinical efficacy against GD2-expressing tumors such as neuroblastoma (1, 11). Anti-*O*-acetylated GD2 mAbs are specific for the *O*-acetylated derivative of GD2, which also is expressed by GD2-positive tumors but, unlike GD2, not by peripheral nerve fibers. Anti-*O*-acetylated GD2 mAbs maintain ADCC activity against GD2-expressing tumors but limit complement activation and, accordingly, decrease neurotoxic side effects. One of these anti-*O*-acetylated GD2 mAbs, mAb 8B6, is able to inhibit the growth of GD2-expressing tumor cells with anti-tumor activity comparable to that of anti-GD2 mAbs.

#### Current Research and Clinical Utility

Table 2 summarizes currently ongoing studies of anti-GD2 mAbs in solid tumors listed in ClinicalTrials.gov.

**Table 2:** Currently ongoing clinical trials of anti-GD2 mAbs.

**Tumor(s)**	**Study description and status**
**ch14.18 (dinutuximab)**	
Neuroblastoma	Phase I study of ^131^I-MIBG + dinutuximab to determine a recommended phase II pediatric dose; currently recruiting; last updated September 2018 (NCT03332667)
Neuroblastoma	Phase II study to evaluate dinutuximab + cytokines (GM-CSF and IL-2) in high-risk patients; active, not recruiting; last updated July 2018 (NCT02169609)
Neuroblastoma	Phase I study of lenalidomide + dinutuximab ± isotretinoin for relapsed/refractory neuroblastoma; active, not recruiting; last updated October 2019 (NCT01711554)
Neuroblastoma	Phase I study to determine the maximum tolerated dose of expanded NK cells + dinutuximab in relapsed/recurrent neuroblastoma; recruiting; last updated May 2019 (NCT02573896)
Neuroblastoma	Phase II study of irinotecan + temozolomide + dinutuximab ± eflornithine in relapsed/refractory neuroblastoma; recruiting; last updated September 2019 (NCT 03794349)
Neuroblastoma	Phase II study of dinutuximab + sargramostim + combination chemotherapy in newly diagnosed high-risk neuroblastoma undergoing stem cell transplant; recruiting; last updated October 2019 (NCT03786783)
Osteosarcoma	Phase II study of dinutuximab + sargramostim in recurrent osteosarcoma; active, not recruiting; last updated October 2019 (NCT02484443)
**hu14.18K322A**	
Neuroblastoma	Phase II study of therapy for children with advanced stage neuroblastoma; last updated October 2019 (NCT01857934)
Neuroblastoma, osteosarcoma, melanoma, Ewing family of tumors	Observational study of pretreatment anti-therapeutic antibodies (anti-hu14.18K322A antibodies as detected by the human anti-human antibodies [HAHA] test); last updated January 2020 (NCT02159443)
**hu3F8**	
Neuroblastoma	Phase I study of humanized anti-GD2 antibody hu3F8 and allogeneic natural killer cells for high-risk neuroblastoma; last updated October 2019 (NCT02650648)
Neuroblastoma	Phase II study of naxitamab (hu3F8) for high-risk neuroblastoma patients with primary refractory disease or incomplete response to salvage treatment in bone and/or bone marrow; last updated January 2020 (NCT03363373)
Neuroblastoma	Phase I/II study of combination therapy of antibody hu3F8 with GM-CSF in patients with relapsed/refractory high-risk neuroblastoma; last updated November 2019 (NCT01757626)
Neuroblastoma	Phase II study of monoclonal antibody 3F8 and sargramostim in treating patients with neuroblastoma; last updated October 2019 (NCT00072358)
Osteosarcoma	Phase II study of humanized monoclonal antibody 3F8 (hu3F8) with GM-CSF in the treatment of recurrent osteosarcoma; last updated January 2020 (NCT02522786)
**3F8**	
Neuroblastoma	Phase I study of beta-glucan and monoclonanal antibody 3F8 in treating patients with metastatic neuroblastoma; last updated February 2019 (NCT00492167)
Neuroblastoma	Phase 2 study of ^131^I-mAb 3F8 in treating patients with central nervous system cancer or leptomeningeal cancer; last updated October 2019 (NCT00445965)
**ch14.18/CHO (dinutuximab beta)**	
Neuroblastoma	Phase I study of investigational medicinal products in children with relapsed/refractory neuroblastoma; last updated July 2019 (NCT02914405)
Neuroblastoma	Phase III study of high-risk neuroblastoma 1.8 of SIOP-Europe (SIOPEN); last updated June 2018 (NCT01704716)

*GM-CSF, granulocyte-macrophage colony-stimulating factor; IL-2, interleukin-2; mAb, monoclonal antibody; MIBG, metaiodobenzylguanidine; NK, natural killer*.

##### Neuroblastoma

Targeted therapy with anti-GD2 mAbs has transformed the management of neuroblastoma. Cheung et al. reported the results of the first study of an anti-GD2 mAb (3F8) in eight patients with this tumor; two achieved a complete response, another two achieved stable disease, and four experienced progressive disease as best outcome (32). It was later observed that the response to treatment in patients with neuroblastoma was improved when GM-CSF, IL-2, and the biologic disease modifier isotretinoin (13-cis-retinoic acid, a retinoid derivative of vitamin A) are combined with anti-GD2 mAbs (73, 85, 86) to enhance ADCC (68). GM-CSF may enhance anti-tumor effects through direct activation and increased numbers of macrophages and granulocytes, and IL-2 is believed to activate NK cells, generating lymphokine-activated NK cells that augment ADCC (87). However, the results of a recent study failed to show evidence that the concurrent administration of IL-2 and the anti-GD2 mAb dinutuximab beta “improved outcomes in high-risk neuroblastoma patients who had responded to standard induction and consolidation treatment” (81). In addition, “IL-2 with dinutuximab beta was associated with greater toxicity than dinutuximab beta alone.” It also should be noted that a clinical study comparing anti-GD2 mAb alone with anti-GD2 mAb plus GM-CSF has not been conducted. A study by Agarwal et al. found G-CSF to be a “tumorigenic growth factor for neuroblastoma” by promoting the expansion of cancer stem cell-like subpopulations *in vitro* and *in vivo*, and the authors suggested “a comprehensive re-evaluation of the clinical use of G-CSF in these patients” (88).

Zhang et al. determined that either anti-GD2 mAb or cytokine-induced killer (CIK)/NK cells isolated from cord blood mononuclear cells significantly provoked apoptosis of neuroblastoma cell line SK-N-SH compared with controls, as assessed by flow cytometry (89). The authors also showed that anti-GD2 mAb and CIK/NK cells in combination significantly increased apoptosis compared with either treatment separately *in vitro*. A currently ongoing clinical trial (ClinicalTrials.gov Identifier NCT02573896) is testing *ex vivo*-expanded NK cells combined with dinutuximab for treatment of children with refractory or recurrent neuroblastoma, based on *in vivo* (mouse model) data published by Liu et al. in 2013 (90).

Disialoganglioside can serve as a prognostic marker as well as a biomarker for neuroblastoma. Balis et al. found that disialoganglioside GD2 was not elevated in children with 10 other childhood cancers except for medulloblastoma (median: 34 nM; range: 6–111 nM) (29). The authors then measured disialoganglioside GD2 in pretreatment serum or plasma samples from 128 children with neuroblastic tumors (ganglioneuroma, ganglioneuroblastoma, and neuroblastoma, 73 of which were high-risk), 8–12 each with 10 other common childhood cancers, and 40 without cancer (controls) (91). The median concentration of GD2 in children with high-risk neuroblastoma was 167 nM (range: 16.1–1,060 nM), which was 30-fold higher than the median concentration (5.6 nM) in controls (*P* < 0.00001). Notably, disialoganglioside GD2 was not elevated in serum from children with differentiated neuroblastic tumors or with other childhood cancers. The authors concluded that disialoganglioside GD2 may be a sensitive and specific circulating biomarker for high-risk neuroblastoma.

Terzic et al. analyzed the expression of disialoganglioside GD2 using immunohistochemical staining prior to targeted therapy with anti-GD2 mAb ch14.18 in 152 tumor samples from patients with neuroblastomas (92). GD2 was expressed in 96% of samples, although the percentage of GD2-positive tumor cells varied across samples. There was an association between a low proportion of GD2-positive cells prior to immunotherapy with ch14.18 and relapse, and the sensitivity of neuroblastoma cell lines to NK cell-mediated lysis varied with the proportion of GD2-positive cells. The results suggested that the proportion of neuroblastoma cells expressing disialoganglioside GD2 in the presence of ch14.18 might be predictive of efficacy.

The U.S. National Cancer Institute was primarily responsible for the development of ch14.18 and sponsored the initial clinical studies over a 20-year period (93–96). This culminated in the pivotal phase III ANBL0032 trial conducted by the Children's Oncology Group (COG) (86). The study included 226 pediatric patients at a median age of 3.8 years (range: 11 months−15 years) with high-risk neuroblastoma who had completed induction therapy, autologous hematopoietic stem cell transplantation (HSCT), and radiotherapy and had achieved at least a partial response before HSCT. Patients were randomized 1:1 to receive up to five cycles of dinutuximab in combination with GM-CSF, IL-2, and isotretinoin followed by a single cycle of isotretinoin alone in the experimental arm or up to 6 months of isotretinoin alone in the control arm. The primary endpoint was event-free survival, defined as the time from randomization to first relapse, progressive disease, secondary malignancy, or death. At interim analysis, there was improvement in event-free survival in patients treated with dinutuximab combination therapy [hazard ratio (HR): 0.57; 95% confidence interval (CI): 0.37–0.89; *P* = 0.01]. Overall survival at 2 years also demonstrated an improvement for patients treated with dinutuximab (HR: 0.58; 95% CI: 0.37–0.91). At the time of the interim report, median overall survival had not been reached in either arm. An updated survival analysis with a median follow-up of 10.3 years (range: 2.3–10.7) found that 73% of patients treated with the dinutuximab combination were still alive compared with 58% of patients treated with isotretinoin alone. Based on these interim results, the COG Data Monitoring Committee recommended terminating randomization, and patients who were previously randomized to receive isotretinoin alone were allowed to cross over to dinutuximab. Notable common adverse events (≥25%) in patients enrolled in the dinutuximab arm of the trial included increased alanine aminotransferase and aspartate aminotransferase, anemia, capillary leak syndrome, diarrhea, infusion reactions, neutropenia, thrombocytopenia, pyrexia, and vomiting. Dinutuximab also caused severe neuropathic pain, and treatment with intravenous opioids was necessary before, during, and after dinutuximab infusion. Morphine administered by nurse- or patient-controlled analgesia is effective for managing the transient nociceptive, generalized pain accompanying dinutuximab therapy (97).

The randomized phase II ANBL1221 trial conducted by the COG compared the combination of irinotecan, temozolomide, and temsirolimumus with irinotecan, temozolomide, and dinutuximab plus GM-CSF in children with relapsed or refractory neuroblastoma (98). The primary endpoint was “best overall response based on results of CT/MRI imaging, MIBG scans and bone marrow aspirates/biopsies, determined after completion of six cycles of protocol therapy.” The authors stated that “irinotecan-temozolomide-dinutuximab met protocol-defined criteria for selection as the combination meriting further study whereas irinotecan-temozolomide-temsirolimus did not. Irinotecan-temozolomide-dinutuximab shows notable anti-tumor activity in patients with relapsed or refractory neuroblastoma.”

Mechanisms of resistance to anti-GD2 mAbs have not been characterized adequately (75), and the reasons for treatment failure have not been identified (99). However, the relatively low response rate observed in the pivotal ANBL0032 phase III trial could be associated with “immune escape mechanisms” utilized by neuroblastoma, including the incursion of immunosuppressive cells, production of abnormal constituents of antigen processing machinery, elaboration of local immunosuppressive factors, and modification of cancer cell metabolism (100). Progression of neuroblastoma during or following treatment with anti-GD2 mAbs also may result from insufficient tumor cell exposure to the antibody, a response by immune effector cells that is inadequate to induce ADCC or CDC, or inherent resistance of the patient's neuroblastoma cell line to antibody therapy (75). Nevertheless, a Cochrane Database Systematic Review published in April 2019 concluded that, based on the results of the pivotal phase III ANBL0032 trial, the evidence base favors “dinutuximab-containing immunotherapy compared to standard therapy concerning overall survival and event-free survival in people with high-risk neuroblastoma pre-treated with autologous HSCT” (101).

##### Small cell lung cancer

Cheresh et al. demonstrated the presence of disialoganglioside GD2 on SCLC-derived tissue and established that the addition of anti-GD2 mAb 14.18 lysed SCLC tumor cells (36). Subsequently, Yoshida et al. showed that addition of anti-GD2 mAbs to cultures of SCLC cells expressing high levels of GD2 and GD3 resulted in marked suppression of growth of GD2-expressing cells and enhanced the apoptotic effect of anti-cancer drugs against SCLC (17). Aixinjueluo et al. suggested that apoptosis of SCLC cells induced by anti-GD2 mAbs may result from the dephosphorylation of focal adhesion kinase, a cytoplasmic tyrosine kinase with anti-apoptotic activity (102).

The DISTINCT study is a recently completed clinical trial of an anti-GD2 mAb in SCLC (ClinicalTrials.gov Identifier NCT03098030). It is a two-part open-label, randomized, unblinded, parallel assignment phase II/III study comparing second-line dinutuximab and irinotecan with irinotecan alone in patients with relapsed or refractory SCLC who progressed following first-line platinum-based therapy (103). In Part 1 of the study, the dose-escalation phase, 12 patients were treated with intravenous dinutuximab in combination with irinotecan (350 mg/m^2^) on Day 1 of 21-day cycles. The combination of dinutuximab and irinotecan was well-tolerated at a median dinutuximab dose of 14 (range: 10–16) mg/m^2^, with no unanticipated adverse events (AEs). AEs of pain were predominantly Grade 1 and did not result in dinutuximab dose reductions, discontinuations, or hospitalizations. Topline results from part 2 of the study, which was designed to determine whether the combination of dinutuximab and irinotecan prolongs overall survival compared with irinotecan alone, did not meet its primary efficacy objective (104).

##### Osteosarcoma

Butch et al. used positron emission tomography (PET) to evaluate the ability of a novel radiolabeled, humanized anti-GD2 mAb ([^64^Cu]Cu-Bn-NOTA-hu14.18K322A) to detect GD2 expression in a mouse model of osteosarcoma *in vivo* (52). Tumor uptake of the radiolabeled mAb was 7-fold higher in modestly GD2-expressing osteosarcomas than in a GD2-negative tumor, and PET scan could identify lesions as small as 29 mm^3^ in a 34% GD2-positive model of metastatic osteosarcoma of the lung. These results support the utility of disialoganglioside GD2 imaging with PET to measure GD2 expression in osteosarcoma and thus maximize the clinical impact of anti-GD2 targeted therapy.

There are currently eight ongoing studies of anti-GD2 targeted therapy in osteosarcoma listed in ClinicalTrials.gov.

##### Soft tissue sarcoma

Previous studies of hu14.18 mAb–IL2 fusion protein and of ^131^I-3F8 mAb were open to various tumor types including sarcoma and completed in 2005, but no results are publicly available and studies no longer are being conducted.

##### Melanoma

Tsao et al. showed that anti-GD2 mAb 3F8 induces apoptosis of GD2-expressing melanoma cells through caspase 3-, 7-, and 8-dependent pathways, downregulation of the anti-apoptotic molecules survivin and cytochrome c, and caspase 9-independent apoptosis-inducing factor release from mitochondria. In addition, analyses of signaling pathway components demonstrated that mAb 3F8 strongly inhibited protein kinase B (Akt) and focal adhesion kinase activation and increased expression of cleaved PARP (105). There are three ongoing clinical studies of anti-GD2 mAbs in patients with metastatic melanoma (one study of ^131^I-3F8 mAb and two of mAb hu14.18K322A) listed in ClinicalTrials.gov.

### Other Anti-GD2 Targeted Therapies

Although many patients with neuroblastoma have responded dramatically to treatment with anti-GD2 mAbs, ~50% will relapse and not survive, and 20% do not respond to initial induction therapy following diagnosis and therefore may not be candidates for subsequent anti-GD2 mAbs under current treatment protocols (106). Other approaches to treatment that target disialoganglioside GD2 are needed for these patients. Anti-GD2 mAbs can be enhanced by conjugation with drugs, cytokines, NK cells, or radiolabeled compounds to deliver a potentially cytotoxic payload to tumors (Figure 2).

#### Anti-GD2 CAR-T Cells

Anti-GD2 chimeric antigen receptor T cells (CAR-T cells) can be engineered to recognize and target disialoganglioside GD2 on neuroblastoma cells, with the potential for greater durability and potency than anti-GD2 mAbs. CAR-T cells also have the ability to cross the blood–brain barrier, which may provide a clinical benefit against gliomas, but have been associated with severe central nervous system (CNS) toxicity involving T-cell infiltration into brain regions known to contain disialoganglioside GD2 (68, 106, 107). In neuroblastoma, initial clinical trials of first-generation anti-GD2 CAR-T cells, constructed primarily of Epstein Barr Virus-specific cytotoxic T lymphocytes with the scFv derived from dinutuximab (14g2a), produced some objective clinical responses (106). However, these responses were not reproduced by second-generation anti-GD2 CAR-T cells (68). Richman et al. compared the antitumor activity of anti-GD2 CAR-T cells derived from 14g2a with that of anti-GD2 CAR-T cells derived from the E101K mutation of GD2 scFv in a human neuroblastoma xenograft in mice. Although the GD2-E101K mutation has enhanced antitumor activity *in vivo*, it was associated with extensive neuronal destruction in the mouse brain and encephalitis localized to the cerebellum and basal regions, which express GD2 in small quantities (107). GD2-specific CAR and interleukin (IL)-15-expressing autologous NK T-cells currently are being studied in a phase I trial in children with relapsed or refractory high-risk neuroblastoma (ClinicalTrials.gov Identifier NCT03294954).

Mount et al. reported that patient-derived glioma cell cultures uniformly express disialoganglioside GD2 and found that anti-GD2 CAR-T cells generated the antigen-dependent cytokines interferon-γ and IL-2 and caused the death of diffuse midline glioma cells *in vitro* (108). Although anti-GD2 mAbs do not cross the blood–brain barrier, anti-GD2 CAR-T-cell therapy is being evaluated in two studies of patients with glioma. The first study (ClinicalTrials.gov Identifier NCT04099797) is scheduled to enroll 34 pediatric patients with high-grade GD2-expressing gliomas or diffuse intrinsic pontine gliomas, whereas the second study (ClinicalTrials.gov Identifier NCT03252171) has enrolled 60 adult patients with GD2-positive gliomas. Anti-GD2 CAR-T cells have also been studied in GD2-expressing Ewing sarcoma (109), glioblastoma (56), and retinoblastoma (62) and are being studied in osteosarcoma (ClinicalTrials.gov Identifier NCT02107963). Brown and Gargett are conducting a phase I study in Australia of the safety and immune effects of an escalating dose of autologous GD2 CAR-T cells in patients with GD2-expressing metastatic melanoma (110). Andersch et al. recently demonstrated *in vitro* that disialoganglioside GD2 is an effective target for CAR-T cell therapy in retinoblastoma (62). CAR-T cell therapy killed all 11 GD2-expressing cell lines studied. Seitz et al. reported that GD2-directed immunotherapy by GD2-CAR-T can prevent metastasis in a highly aggressive mouse model of triple negative breast cancer (111).

#### GD2 Vaccines

The goal of vaccine-based immunotherapy is to produce a specific anti-tumor response directed against a specific tumor-associated antigen such as disialoganglioside GD2 (112). However, because most tumor-associated antigens are “self” antigens (i.e., idiotypic), presence of pre-existing immune tolerance against such idiotypic antigens is a barrier to effective active immunization with an idiotypic vaccine. On the other hand, anti-idiotypic vaccines utilize anti-idiotypic mAbs as surrogate antigens to avoid this problem and induce humoral and/or cellular immune antitumor responses against non-protein tumor antigens. Although several preclinical studies using anti-idiotypic antibodies supported their utility as anti-tumor vaccines, human studies have been disappointing and anti-idiotypic vaccines have failed in later phase trials (113). Bivalent vaccines containing neuroblastoma-associated antigens GD2 and GD3 together with the immunologic adjuvant OPT-821 have generated anti-GD2 and anti-GD3 IgG antibody responses in patients with neuroblastoma and currently are being studied in phase II trials (66, 114).

Carvajal et al. reported the results of a randomized, placebo-controlled phase II trial in which 136 patients with stage IV sarcoma received a trivalent vaccine against GD2, GD3, and GM2 or placebo plus the immunologic adjuvant OPT-821 (ClinicalTrials.gov Identifier NCT01141491) (115). At entry, 90% of patients had relapsed disease and 14% had ≥4 metastases resected. Induction of high IgG and IgM titers was observed in 52 and 24% of patients, respectively, receiving vaccine compared with 0 and 2% receiving placebo, but there was no significant difference in progression-free survival between the two groups.

#### Novel Anti-GD2 Therapies

In 2014, Ahmed and Cheung published a comprehensive review of novel approaches to anti-GD2 mAb-based immunotherapy for cancer, with the objectives of reducing tumor burden and curing neuroblastoma patients and patients with other solid tumors while lowering the dose and limiting the intensity of cytotoxic chemotherapy and radiotherapy (74). Cytokines are added to antibody treatment regimens to augment effector cell function but can also produce dose-limiting systemic side effects (116). Conjugation, or fusion, of cytokines to mAbs (immunocytokines) may avoid this issue, as, for example, Hu14.18-IL-2 anti-GD2 immunocytokine (116, 117). Other novel anti-GD2 therapies include targeted nanoparticles (presenting anticancer drugs at a sustained rate directly to tumor cells) (118), antibody–drug conjugates (allowing chemotherapeutic agents and other toxic drugs to be released into the microenvironment of the tumor via antibody targeting) (119), T-cell engaging bispecific antibodies (binding to two different antigens) (120), radiolabeled antibodies (delivering radiotherapy directly to the tumor environment) (121), and cell surface expression of chimeric antigen receptors (merging specific antigen binding with T-cell effector functions) (122). Barry et al. determined that the combination of dinutuximab and adoptively transferred *ex vivo*-activated human NK cells significantly improved the survival of immunodeficient mice following resection of neuroblastoma xenografts compared with dinutuximab or activated NK cells alone (123).

## Summary and Conclusions

Unlike other gangliosides, which are expressed by most normal tissues, disialoganglioside GD2 expression is restricted to the CNS, peripheral sensory nerve fibers, dermal melanocytes, lymphocytes, and mesenchymal stem cells. Disialoganglioside GD2 is also expressed uniformly and abundantly by the vast majority of neuroblastomas, most melanomas and retinoblastomas, and many Ewing sarcomas, whereas its expression by SCLC, gliomas, osteosarcomas, and soft tissue sarcomas is more variable. Because it is expressed across a relatively broad range of tumor types, disialoganglioside GD2 can be considered a tumor-associated antigen and may promote a more malignant tumor phenotype through enhanced cell proliferation, growth, motility, migration, adhesion, and invasion. The expression profile of disialoganglioside GD2 and its role in cancer biology therefore provide a justification and rationale for clinical targeting of this antigen with anti-GD2 mAbs and other therapeutic approaches. Anti-GD2 mAbs ch14.18 and ch14.18/CHO have been approved for the treatment of neuroblastoma. Clinical trials should clarify the role of anti-GD2 therapy in disialoganglioside GD2-expressing malignant tumors.

## Author Contributions

Conception by TO. Manuscript development, writing, and approval by TO, CI, and BN. All authors contributed to the article and approved the submitted version.

## Conflict of Interest

TO declares research funding (to institution) from United Therapeutics Corp. The remaining authors declare that the research was conducted in the absence of any commercial or financial relationships that could be construed as a potential conflict of interest.
